# On the Natural History of Flavin-Based Electron Bifurcation

**DOI:** 10.3389/fmicb.2018.01357

**Published:** 2018-07-03

**Authors:** Frauke Baymann, Barbara Schoepp-Cothenet, Simon Duval, Marianne Guiral, Myriam Brugna, Carole Baffert, Michael J. Russell, Wolfgang Nitschke

**Affiliations:** ^1^CNRS, BIP, UMR 7281, IMM FR3479, Aix-Marseille University, Marseille, France; ^2^Jet Propulsion Laboratory, California Institute of Technology, Pasadena, CA, United States

**Keywords:** electron bifurcation, redox cooperativity, flavoenzymes, emergence of life, redox enzyme construction kit, bioenergetics

## Abstract

Electron bifurcation is here described as a special case of the continuum of electron transfer reactions accessible to two-electron redox compounds with redox cooperativity. We argue that electron bifurcation is foremost an electrochemical phenomenon based on (a) strongly inverted redox potentials of the individual redox transitions, (b) a high endergonicity of the first redox transition, and (c) an escapement-type mechanism rendering completion of the first electron transfer contingent on occurrence of the second one. This mechanism is proposed to govern both the traditional quinone-based and the newly discovered flavin-based versions of electron bifurcation. Conserved and variable aspects of the spatial arrangement of electron transfer partners in flavoenzymes are assayed by comparing the presently available 3D structures. A wide sample of flavoenzymes is analyzed with respect to conserved structural modules and three major structural groups are identified which serve as basic frames for the evolutionary construction of a plethora of flavin-containing redox enzymes. We argue that flavin-based and other types of electron bifurcation are of primordial importance to free energy conversion, the quintessential foundation of life, and discuss a plausible evolutionary ancestry of the mechanism.

## Introduction

Life fundamentally depends on the free energy (ΔG) provided by the electrochemical disequilibrium between reduced, electron-donating and oxidized, electron accepting environmental substrates (see Schoepp-Cothenet et al., 2013). One might think that metabolic redox reactions must thus occur within the redox span imposed by these substrates. This clearly is not the case. The acidophilic iron oxidizers from the Acidithiobacillaceae for instance provide a rather extreme counterexample to such redox range limitations. These organisms use Fe^2+^ (with E_m_ >>0 mV) as environmental reductant but nonetheless manage to maintain their NAD and ferredoxin pools (featuring E_m_s below -300 mV) partially reduced. The necessary up-conversion of redox energy in these species (Ingledew, 1982; Ferguson and Ingledew, 2008; reviewed in Nitschke and Bonnefoy, 2016) as well as in a multitude of other organisms facing the same problem is carried out with the help of the chemiosmotic membrane potential which serves to accumulate incremental ΔG from several individual redox reactions. While this scheme is often referred to as “driving electrons uphill,” a more appropriate view is that it uplifts the reducing power of these electrons so that they can flow downhill toward NAD and ferredoxins.

This kind of chemiosmotically driven “reverse electron transfer” was considered the general and unique mechanism to augment the reducing power of environmentally provided reducing equivalents to the levels demanded by metabolic needs. That is, until flavin-based electron bifurcation entered the scene. In 2008, the enzyme ETF (electron-transfer-flavoprotein) was reported to singlehandedly, i.e., in one redox reaction and without the implication of a chemiosmotic membrane system, be able to reduce electron acceptors having electrochemical potentials substantially more reducing than that of the electron donating substrate (Herrmann et al., 2008; Li et al., 2008). Reduction of the low redox potential acceptor, however, was observed to be dependent on the presence and concomitant reduction of a second substrate with much higher redox potential. Likewise, reduction of the high potential substrate was found to depend on the presence and reduction of the low potential acceptor (Chowdhury et al., 2014, 2015, 2016; Demmer et al., 2017). Enzyme-bound flavin, a two-electron redox compound, was proposed as the crucial redox cofactor permitting the coupling between an endergonic one-electron transfer reaction to the low potential acceptor and a (more strongly) exergonic one reducing the high potential acceptor (Chowdhury et al., 2016). The two electrons on the fully reduced flavin are thus thought to bifurcate toward energetically dissimilar pathways heaving one electron onto a more strongly reducing state while still obeying the thermodynamic imperative of a negative ΔG for the entire two-electron redox reaction (Buckel and Thauer, 2013). Since the first proposal of flavin-based electron bifurcation (Herrmann et al., 2008), a multitude of electron bifurcating enzymes containing flavins have been reported to operate in diverse types of redox metabolisms (for reviews see Peters et al., 2016; Buckel and Thauer, 2018a,b; as well as the most recent member of the family described in Duan et al., 2018).

The conceptual similarity of flavin-based electron bifurcation to the so-called Q_o_-site reaction in the membrane-integral Rieske/cyt*b* complexes (alias Complex III, *bc*_1_, *b*_6_*f*, etc.) was pointed out early on (Herrmann et al., 2008). The core functional reaction in Peter Mitchell’s Q-cycle mechanism (Mitchell, 1975, 1976) consists in the coupled redox transitions between a quinone, i.e., a two-electron redox compound, bound at the Q_o_-site of the enzyme and a pair of one-electron redox centers one of which features a higher and the other one a lower electrochemical potential than the quinone (see also Wikström and Berden, 1972). The fundamental electrochemical and structural properties allowing the Q_o_-site reaction to occur have been worked out over several decades (for comprehensive reviews, see Osyczka et al., 2004; Crofts et al., 2013) and part of this contribution will be dedicated to further explore common features of these two types of electron bifurcation to progress toward a comprehensive molecular and thermodynamic understanding of the flavin-based case (see also Nitschke and Russell, 2012; Peters et al., 2016; Buckel and Thauer, 2018a). At the end of this contribution we will discuss the potential of the electron bifurcation reaction to contribute to the emergence of life on earth.

## The Peculiarities of Two-Electron Redox Compounds and the Concept of Redox Cooperativity

As worked out almost a century ago (Michaelis, 1932), certain heteronuclear aromatic two-electron redox compounds (and most prominently quinones and flavins) can exhibit astonishingly versatile redox properties. Under certain conditions they undergo two consecutive redox transitions between the oxidized (ox) and the one-electron- (semi-) reduced and between the 1-electron- and the 2-electron “fully” reduced (red) states. In this case, the redox midpoint potentials of the two individual transitions follow the intuitive rule that putting the second electron on the molecule will be “harder” (i.e., occur at a lower electrochemical potential) than adding the first one. However, under appropriate conditions in protic solvents, the first transition toward the semi-reduced state can render, due to redox-coupled protonation/hydrogen-bonding events (Lim et al., 2013; Tan et al., 2015), the second transition more favorable than the first one so that both electrons will come or go virtually together and the observed redox transition will follow an *n* = 2-type behavior^1^. A redox transition where both electrons are transferred together is commonly called “cooperative.” More generally, the terms “positive” and “negative cooperativity” are used to distinguish the “both together” from the “one-by-one” redox regimes. Negative cooperativity means that the first reduction reaction leads to a decrease in the midpoint potential for the second reduction (i.e., corresponding to the “intuitive” situation mentioned above) and *vice versa*. As shown by Michaelis (1932), negatively and positively cooperative two-electron redox transitions form a continuum characterized by a smoothly varying spacing ΔE between the redox midpoint potentials of the individual transitions ΔE = E_ox/sr_ – E_sr/red_.

**FIGURE 1:**
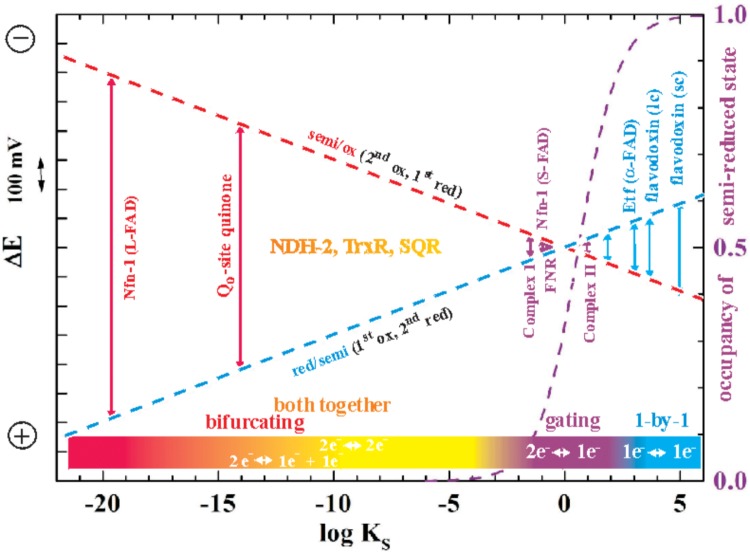
Redox seesaw-type representation of the correlation between the electron transfer function of selected flavoenzymes and the redox cooperativity of their flavin cofactors. The redox midpoint potentials of the first (oxidized to one-electron reduced, red dashes) and the second redox transition (half-reduced to two-electron reduced, blue dashes) is shown as a function of the semiquinone stability constant K_S_ = [semi-reduced]^2^/[ox]x[red]. The maximal occupancy of the flavosemiquinone as would be observed in equilibrium redox titrations on the respective flavins is indicated as a violet dashed curve. Note that in this and all following figures more negative potentials are toward the upper part of the graphs.

While of course strong negative cooperativity implies that in equilibrium redox titrations the semi-reduced form is stable over a wide range of ambient potentials, increasingly positive cooperativity will be accompanied by a decreasing stability constant K_S_^2^ of this semi-reduced state translating into lower percentages of the semi-reduced state observable at equilibrium. The relationship between K_S_ and ΔE is most clearly illustrated by the so-called “redox seesaw” representation (Mitchell, 1976)^3^. **Figure 1** illustrates the relationship between the redox midpoint potentials of the individual redox transitions and K_S_. For K_S_ < 1 (logK_S_ < 0), E_2_ is more positive than E_1_. The logK_S_ < 0 domain is therefore frequently referred to as the region of “inverted” or “crossed-over” potentials.

## The Flavin, a Redox Cofactor for All Seasons

As indicated in **Figure 1**, the specific binding sites in enzymes confer very diverse degrees of cooperativity to the flavin cofactors. The resulting differences in redox behavior allow these two-electron redox compounds to be used by life for a plethora of different purposes. The strictly one-by-one-type flavodoxins thus play the role of one-electron transport proteins with, in general, only one of the two redox transitions being physiologically relevant. A well-studied example are the flavodoxins found in certain cyanobacteria under iron-limiting growth conditions. In these systems, the semi-reduced/reduced transition serves to accept electrons from Photosystem I and to distribute them to the physiological electron acceptors just as [2Fe-2S]-ferredoxins would do under iron-replete conditions (Sétif, 2001). By contrast, flavin cofactors mediating two-electron redox reactions between substrates with strong positive redox-cooperativity, such as NADH and quinone, function optimally if they feature strongly destabilized semi-reduced states, i.e., if they are far into the region of inverted potentials shown in **Figure 1**. The flavoenzyme NDH-2 for example, a single-polypeptide NADH:quinone oxidoreductase associated to the n- (i.e., negatively charged) side of bioenergetic membranes mediates the two-electron transfer involved in the oxidation of NADH and the reduction of quinone. It does not involve observable semi-reduced states of the flavin (de Vries and Grivell, 1988). Finally, two-electron redox compounds with redox properties in the vicinity of logK_S_ = 0 in **Figure 1** strike a compromise between strictly one- and strictly two-electron transitions and hence function as “gates” mediating between 2-electron and 1-electron segments of electron transfer chains. A prominent example is provided by FNR (ferredoxin NAD(P)^+^ reductase) which deals with both one-electron (ferredoxin) and two-electron (NAD) redox partners (Beirns and Wang, 1972; Batie and Kamin, 1981; Sánchez-Azqueta et al., 2014). In **Figure 1** we distinguish the mentioned types of electron transfer reactions by color-coding “single-electron” reactions in blue, “gating”-type ones in violet and “both-together” ones in orange/red. So where does electron bifurcation fit into this scheme?

## A Common Scheme for Quinone- and Flavin-Based Electron Bifurcation

**FIGURE 2:**
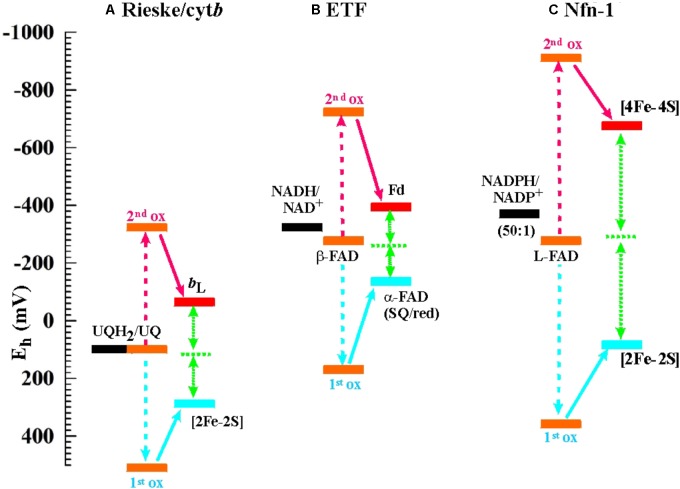
Electrochemical landscapes for the electron bifurcation reactions in **(A)** the Rieske/cyt*b* complexes (the shown potentials are for the ubiquinone-oxidizing enzymes in mitochondria and alpha-proteobacteria; for enzymes operating with other types of quinones, see Bergdoll et al., 2016), **(B)** ETF, and **(C)** Nfn-1.

As stated above, flavin-based electron bifurcation likely relies on the same thermodynamic concept as its quinone-based counterpart observed at the Q_o_-site of *bc*_1_-complexes and related enzymes. Research on the Q_o_-site reaction is ahead of the flavin case by several decades and many years of unsuccessful attempts to observe the EPR signature of the semi-reduced state of the quinone in the Q_o_-site of the Rieske/cyt*b* complexes under conditions of equilibrium redox titrations suggested a very low K_S_ value. This led to the conclusion that the Q_o_-site quinone must undergo a two-electron transition with strong positive cooperativity during enzyme turnover in line with Peter Mitchell’s original hypothesis (Mitchell, 1976). More recent quantitative determinations yielded indeed astonishingly low values in the region of logK_S_ = -14 (Zhang et al., 2007; Crofts et al., 2013), that is, even lower than the semiquinone stability in bulk aqueous solutions (logK_S_ = -9 to -10.5 according to the chemical type of quinone, Hauska et al., 1983; Song and Buettner, 2010). The midpoint potentials for the individual one-electron redox transitions corresponding to such low K_S_ values yield a redox landscape for electron transfer through the Rieske/cyt*b* complexes as depicted in **Figure 2A**. Remarkably, the general layout of this redox landscape appears to be conserved in all types of Rieske/cyt*b* complexes analyzed so far (Bergdoll et al., 2016). The most striking feature of this redox scheme is the strongly uphill electron transfer from the quinol/semiquinone couple to the Rieske [2Fe-2S] cluster. This endergonic reaction upends the traditional view which referred to reduction of the Rieske cluster as the exergonic branch of the bifurcating Q_o_-site reaction and the reduction of heme *b*_L_ as the endergonic one. However, the strong endergonicity (ΔE_m_ ∼ 200 mV) of electron transfer from the quinol to the Rieske cluster provides a straightforward rationale for the strong coupling observed for the two redox steps in oxidation of the quinol. While the electron tunneling back and forth between the quinol and the Rieske [Fe-S]-cluster resides more than 99.9% of the time on the quinone, the full two-electron oxidation reaction becomes (slightly) exergonic if oxidation of the semiquinone by the second electron acceptor (heme b_L_) can also occur. The driving force for oxidation of the semiquinone therefore pulls the thermodynamically impeded “first” electron transfer over the activation barrier. This semiquinone, however is of course only present and thus available for oxidation while this “first” electron resides on the [2Fe-2S]-cluster. The probability of this configuration thus determines the kinetics of the coupled two-electron reaction. The combined reaction pattern has been aptly described as an “escapement” mechanism (Crofts et al., 2013) wherein the entire reaction can only occur if the two individual steps are both occurring. The coupling between the individual reactions is imposed by the specific electrochemical properties (i.e., the strongly inverted individual redox midpoint potentials) conferred to the quinone by the Q_o_-binding site. The entire reaction is of course reversible and flavins (or quinones) with crossed-over potentials can be reduced by two electrons from distinct donors with the first reduction step at a lower redox potential than the second one. This process is referred to as electron confurcation and systems proposed to rely on electron confurcation encompass enzymes involved in sulfur metabolism such as QmoA and QmoB (Ramos et al., 2012; Grein et al., 2013) as well as certain hydrogenases (Schut and Adams, 2009; Wang et al., 2013b; Zheng et al., 2014).

Being a relatively recently discovered phenomenon, it is not surprising that knowledge of the electrochemical details regarding flavin-based electron bifurcation is still limited. We have depicted the two systems for which the relevant redox parameters have been obtained, i.e., the enzymes NADH-dependent ferredoxin:NADP^+^ oxidoreductase (Nfn-1) and the bifurcating electron-transfer-flavoprotein (ETF) in **Figures 2B,C** next to that detailed above for the Rieske/cyt*b* complex. It is noteworthy that the exact value for the K_S_ of the bifurcating flavin in ETF has not been determined so far but has been assumed (Chowdhury et al., 2016; Demmer et al., 2017) in analogy to the Nfn-1 results (Lubner et al., 2017) or to the value obtained for the quinone in the Q_o_-site reaction (Osyczka et al., 2004; Crofts et al., 2013). Determination of K_S_ for the bifurcating flavin in Nfn-1 is itself based on an indirect method involving the redox midpoint potential of the [4Fe-4S]-cluster oxidizing the photochemically generated flavo-semiquinone to the fully oxidized state. Redox midpoint potentials of the two [4Fe-4S]-clusters in Nfn-1 have been obtained *via* equilibrium redox titrations (Lubner et al., 2017) and the E_m_ of the cluster accepting the electron from the flavin under conditions of the photochemical experiment are likely to differ from equilibrium titration values as a result of electrostatic interactions in the latter approach. These experimental uncertainties notwithstanding, the bifurcating flavin in ETF can be safely assumed to feature strongly inverted individual redox midpoint potentials since strict two-electron behavior has been observed in equilibrium redox titrations on the enzyme from *Megasphaera elsdenii* (Sato et al., 2013) and on the FixAB-homolog in *Rhodopseudomonas palustris* (Duan et al., 2018).

The comparison of the three redox landscapes depicted in **Figure 2** reveals an intriguing unicity for the overall thermodynamics of the different types of electron bifurcation. In all cases, extracting the first electron from the fully reduced two-electron redox compound turns out to be a strongly endergonic step which needs the second oxidation step to occur in order to become stabilized. Similarly, in all cases the full oxidation of the two-electron redox compound is slightly exergonic but remains very close to thermodynamic equilibrium.

The picture emerging from the comparison of the admittedly small sample of sufficiently well-characterized flavin-based systems (**Figures 2B,C**) to the quinone-based Q_o_-site reaction (**Figure 2A**) is thus that of a common mechanistic principle based on strongly crossed-over electrochemical potentials for the individual redox transitions of the two-electron redox compounds and a strong endergonicity of the first oxidation step yielding all the ingredients required for the escapement-type mechanism discussed above.

Intriguingly, in all quinone- and flavin-based electron bifurcating enzymes for which 3D-information is available, a large-scale conformational movement of a protein domain, in most cases that carrying the electron acceptor for the first electron, has been observed or inferred (Zhang et al., 1998; Demmer et al., 2017; Lubner et al., 2017; Wagner et al., 2017). The above described mechanism *per se* does not present an obvious need for such conformational changes. These conformational movements may serve to optimize electron transfer rates and minimize efficiency-decreasing back- or deleterious side-reactions. A more comprehensive characterization of the flavin-based systems may in the future allow a deeper understanding of the roles of conformational changes in the phenomenon of electron bifurcation.

**FIGURE 3:**
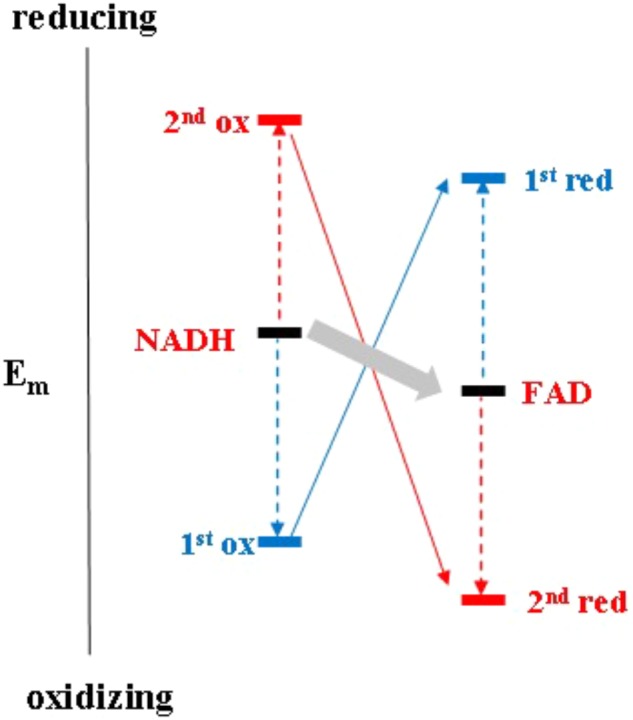
Electrochemical landscape for the two-electron transfer reaction between NADH and FAD in the framework of the escapement-type mechanism discussed in the text.

It is worth noting that electron bifurcation obviously occurs in roughly the same region of inverted redox midpoint potentials as do “both together”-type 2-electron transfer reactions such as for example in NDH-2 (**Figure 1**). This is not a coincidence since two-electron redox compounds react extremely sluggishly with a one-electron donor or acceptor because of their strongly inverted redox midpoint potential. The K_S_ for the NADH/NAD^+^ couple has been determined at 10^-20^ (Anderson, 1980) yielding redox midpoint potentials for the first oxidation of +340 mV and for the second one of -940 mV. Electron acceptors for NADH typically have redox midpoint potentials well below 0 mV and the first oxidation step is therefore steeply uphill. This endergonicity is even dramatically enhanced if the acceptor is a two-electron redox compound which has itself strongly inverted redox midpoint potentials since its 1^st^ reduction potential E_1_ is substantially lower than its average two-electron potential E_m_ (see **Figure 3**). The transfer of the second electron (from -940 mV), however, will occur toward the 2^nd^ redox transition of the acceptor molecule which is, in the case of inverted redox midpoint potentials, higher than the average two-electron potential E_m_. This step will therefore be strongly exergonic and render the combined reaction (gray arrow in **Figure 3**) favorable whereas in the presence of only single-electron acceptors the electron will remain on the NADH molecule. “Both-together” reactions can therefore be regarded as processes which bifurcate the individual electrons thermodynamically, that is, toward strongly dissimilar reduction potentials, but not spatially. The two redox transitions of the two-electron acceptor with dissimilar (crossed-over) redox midpoint potentials play the same role as the two electrochemically AND spatially distinct electron acceptors in electron bifurcation for rendering the total reaction thermodynamically downhill.

## The Importance of Electron Bifurcation Based on Strongly Inverted Redox Potentials to Life

The thermodynamic outcome of electron bifurcation as described above fundamentally differs from that of the vast majority of enzyme-mediated catalyzes. While the latter perform reactions which in general uniformly decrease the free energy of the system (a different way of saying that they are exergonic), electron bifurcation certainly is exergonic overall but it generates a product (one out of two) which is even farther from thermodynamic equilibrium than the educt. In ETF for instance, NADH can reduce flavins exergonically in a two-electron reduction reaction, but the ferredoxin reduced as a result of ensuing flavin-based electron bifurcation is substantially more reducing than the initial reductant, NADH. Extending this reasoning to the thermodynamics of life in its redox context, i.e., its source of environmental free energy (Schoepp-Cothenet et al., 2013), an organism growing for example on molecular hydrogen as electron donor augments, *via* electron bifurcation, the reducing power available to drive metabolic reactions far above the reducing power directly furnished by its habitat. This increase in electrochemical disequilibrium corresponds to a decrease in entropy in the sub-group of redox reactions involving the so-produced reduced ferredoxin molecules. As a pay-back, the other electron emerging from the electron bifurcation reaction features a higher redox midpoint potential resulting in a lower redox disequilibrium between this electron and its acceptor compounds. Electron bifurcations can therefore alternatively be regarded as free energy converters which drive an increase of order in the living system over that of the environment *via* a concomitant generation of “heat,” i.e., the exergonic, entropy-increasing redox reaction.

The essence of electron bifurcation as encountered in ETF, Nfn-1, or the Q_o_-site reaction thus lies in its ability to increase redox energy above that provided by the environment. As detailed above, this necessarily involves strongly inverted redox potentials. Recent articles addressed the question whether electron bifurcation was conceivable without involving strong positive redox cooperativity (Hoben et al., 2017; Zhang et al., 2017). While we do not dispute the general possibility of the proposed scenarios based on uncrossed potentials (Zhang et al., 2017) we would hold that, for the enzymes considered in these articles, such scenarios miss the point since the respective types of electron bifurcation would be useless to biology. In these scenarios the bifurcating cofactor needs to be fully reduced by upstream electron donors with redox potentials commensurate or lower than that of the low potential electron acceptor of the bifurcating reaction. The entropic benefit for the organism would therefore be nil in scenarios based on uncrossed potentials. However, in certain cases a kinetic rather than thermodynamic benefit may be reaped from only spatially bifurcating electrons as we will argue happens in the case of the NuoE/F system of Complex I discussed below. In such circumstances strong inversion of redox midpoint potentials may be dispensable.

## What About Geometry?

Kinetics of bifurcation reactions, like all electron transfer kinetics, depend on several parameters implemented in Marcus’ theory of outer shell electron transfer (Moser et al., 1992). Beside ΔG and reorganization energy, distances between co-factors strongly influence the kinetic details of such electron transfer reactions. For the majority of flavin-based systems crucial information necessary to apply this rigorous formalism is still missing today or is incomplete. For example, in the 3D structures of both ETF and heterodisulfide reductase (Hdr), a number of electron transfer distances to or from the flavin are too long to permit biologically relevant rate constants and large-scale domain movements must therefore be invoked (Demmer et al., 2017; Wagner et al., 2017). This problem is less striking for Nfn-1, although the distance between the bifurcating flavin and the [2Fe-2S]-cluster on the high potential branch at about 14 Å may be close to the upper limit for physiologically sensible electron transfer (Lubner et al., 2017). Still, the attribution of redox midpoint potentials to individual [4Fe-4S]-centers on the low potential branch in Nfn-1 so far is arbitrary and not substantiated by experimental evidence. As mentioned before, these redox midpoint potentials have furthermore been determined through equilibrium titrations and may be lower than “operating” potentials due to electrostatic repulsion effects.

**FIGURE 4:**
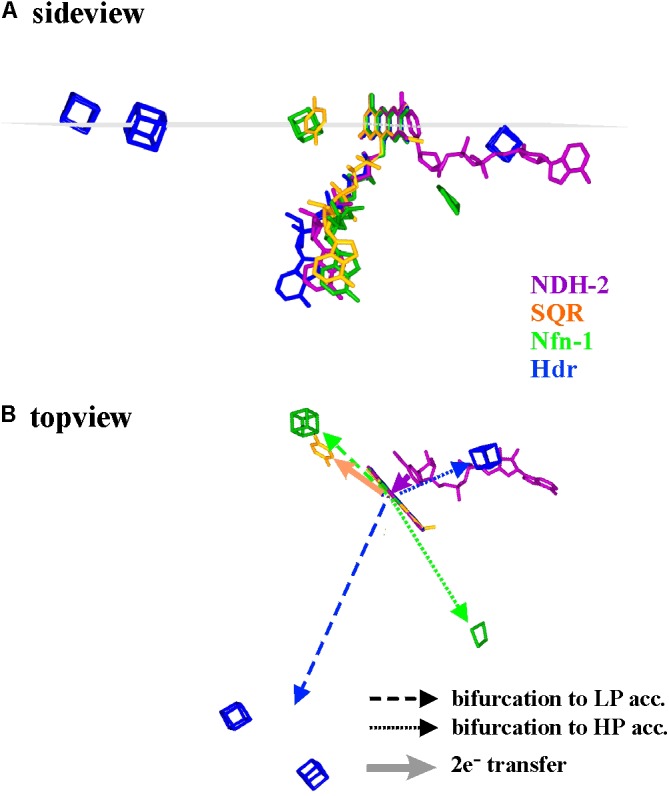
Spatial arrangement with respect to the flavin of electron donating and accepting cofactors in four different flavoenzymes, two of which perform electron bifurcation (Nfn-1 and Hdr), while the other two transfer both electrons together from/to their redox partners. The sideview in **(A)** renders the cofactors as seen from within a plane perpendicular to the flavin macrocycle (indicated in gray) while in **(B)** the same cofactors are seen from above this plane. LP and HP stand for low and high potential electron acceptors, respectively.

As a first approximation, we therefore compared the available 3D-structures in order to assess whether general properties of electron transfer pathways between the bifurcating flavins and their electron donors or acceptors can be found. An exhaustive representation of this comparison is presented as **Supplementary Figure S1** and a few representative examples are shown in **Figure 4**. In this figure, the structural arrangements of electron donors and acceptors with respect to the flavin are depicted from two different view angles for the enzymes Nfn-1, Hdr, NDH-2, and SQR (Sulfide quinone reductase). Since in Hdr the presumable electron acceptor on the low potential branch is one of two roughly equidistant [4Fe-4S]-centers (Wagner et al., 2017), we have shown both of these clusters in **Figure 4**. Furthermore, substantial movement of the domain binding these two centers is likely to occur (Wagner et al., 2017) and the indicated direction of electron transfer is therefore somewhat uncertain. The quinone acceptor was not resolved in the published 3D-structure of NDH-2 and is therefore only indicated for the SQR enzyme. *Vice versa*, the electron donating NADH was only identified in NDH-2.

The shown structural comparison yields the following conclusions. (i) The donor/acceptor substrates NADH/NAD^+^ are accommodated co-planar to the isoalloxazine ring system on the side opposite to the bending direction of the tail leading to the adenine nucleotide, i.e., uncorrelated to the Si- or the Re-side (see **Supplementary Figure S1**). (ii) Other one- and two-electron redox partners (iron–sulfur centers, quinone) are clustered in or near a plane perpendicular to the isoalloxazine ring system and containing its long axis (**Figure 4A** and **Supplementary Figure S1**). (iii) Most single-electron transfer reactions from or to the flavin seem to proceed from the extremities of the conjugated isoalloxazine moiety. Notable exceptions to this rule are provided by Hdr (**Figure 4**, blue cofactors) or Complex I (see below). In all available structures, however, the two electrons involved in bifurcating/confurcating electron transfer reactions take diametrically opposite directions to/from their acceptor/donor molecules (**Figure 4B**).

The comparison shown in **Figure 4** thus indicates that overall electron transfer geometry does not seem to play a significant role in the type of electron transfer the flavin is involved in (gating, both-together, bifurcating). This comforts our view that electron bifurcation is foremost an electrochemical phenomenon.

## The Flavoprotein Construction Kit; Toward a Basic Set of Structural Motifs

**FIGURE 5:**
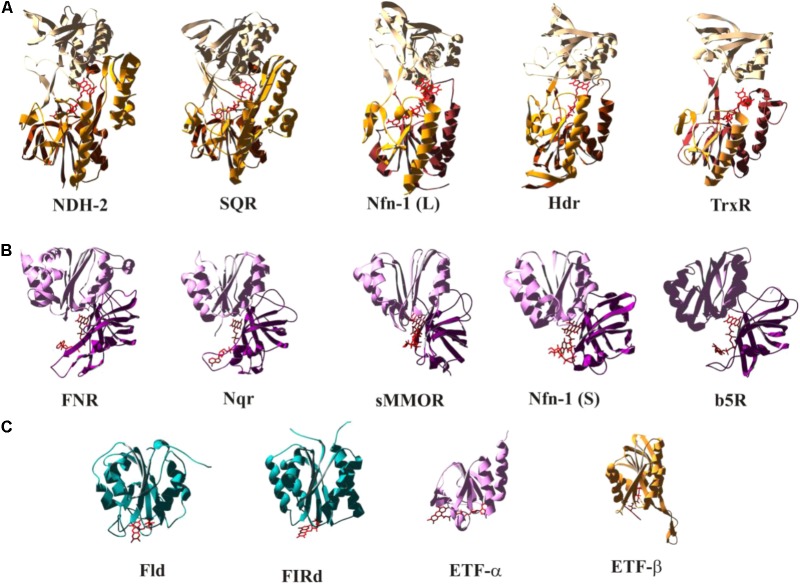
Structural comparison of selected flavoenzymes or their flavin-containing domains highlighting conserved folds as discussed in the text. The color-coding indicates the mode of electron transfer reactions as defined in **Figure 1**. A more extended version of this figure, containing substantially more cases, can be found as **Supplementary Figure S2**. Detailed electrochemical parameters and relevant references are listed in **Supplementary Table S1**.

As illustrated in **Figure 1**, the redox properties of diverse flavoproteins cover the whole range allowed by the electrochemistry of two-electron redox compounds and they correspondingly perform as well “one-by-one” as “one/two gating,” “both together,” and “bi- and confurcating” electron transfer reactions. While the vast majority of flavin-binding domains in proteins are made up from Rossmann-fold-type (Rao and Rossman, 1973) basic units, substantially differing variations to this general theme have been observed. We have tried to classify a number of flavin-binding domains relevant to bioenergetic redox reactions into structural motifs based on their 3D structures and have identified three distinguishable architectures which we denote as (a) the NDH-2-type, (b) the FNR-type, and (c) the flavodoxin-type folds (see **Figure 5**). During our searches of 3D databanks we came across cases which either seemed to be more distantly related to one of these families (**Supplementary Figure S2a**, right hand part and S2a′), to result from convergent evolution or to represent entirely different folds (**Supplementary Figures S2d–f**). We aren’t familiar with these enzymes which are at the border of the field of bioenergetics but have nevertheless included these additional cases in the **Supplementary Figure S2** and listed their electrochemical properties in **Supplementary Table S1**.

The NDH-2- (**Figure 5A**) and the FNR-type (**Figure 5B**) motifs fall into distinct structural domains with the flavin sandwiched in between these domains. In **Figures 5A,B**, the N-terminal domains are at the bottom of the depicted structures followed by domains colored in a lighter shade and represented in the upper part of the individual structures. The NDH-2 folds feature a third domain (marked in bright orange in **Figure 5**) that reaches back down toward the N-terminal domain. The C-terminal domain of the FNR fold (**Figure 5B**, light pink) strongly resembles the flavodoxin fold (**Figure 5C**). The flavodoxin fold can also be identified in pyruvate oxidases (**Supplementary Figure S2g**), in NuoF, a subunit of Complex I (**Supplementary Figure S2h**), and in flavorubredoxin reductase (**Supplementary Figure S2i**). Pyruvate oxidases and flavorubredoxin reductase are composed of two flavodoxin-like domains. NuoF features the flavodoxin domain at its N-terminus followed by a β-sheet and an α-helical domain, whereas in FNR the flavodoxin domain constitutes the C-terminal part of the protein and is preceded by an entirely β-sheet-containing N-terminal domain. The flavodoxin fold therefore appears to have been fused several times independently to various other domains in order to build the above discussed enzymes in a nice illustration of the construction kit principle used by nature to build novel enzymes (Baymann et al., 2003). Sandwiching the flavin moiety within the contact region of two/three domains results in a relatively uniform accommodation of the isoalloxazine ring system among the different systems. The peripheral location of the flavin in flavodoxin-type folds, by contrast, allows for substantial variability in arrangement of the cofactor in this structure (see **Figure 5C**).

Several cases, such as for example HdrA, feature extensive sequence insertions within the flavin-binding units resulting in additional, sometimes cofactor-binding domains while nevertheless maintaining the common overall structure of the flavin-domain. While these somewhat freakish insertions are relatively rare, a sequential lining up of separate domains containing extra cofactors or that are even devoid of such cofactors in a single polypeptide chain frequently occurs. Thus, for the sake of clarity, the structures shown in **Figure 4** feature only the relevant flavin-domains omitting the (sometimes substantial) remainder of the entire protein.

The NuoF case is part of the folds where an extra cofactor, a [4Fe-4S]-cluster is harbored by an additional domain and is positioned at 8 Å distance from the flavin. A [2Fe-2S]-center is furthermore located at a distance of 13 Å on the neighboring NuoE subunit. This structural arrangement thus looks perfectly suited for electron bifurcation as in the cases mentioned above. However, the electrochemical properties determined for the involved cofactors (Gnandt et al., 2016) shows that the NuoE/F system doesn’t follow the scheme depicted in **Figure 2**. In the following we will discuss the fold and the electron transfer properties of this intriguing case in more detail.

## The Puzzling Case of the NuoE/F-Family

**FIGURE 6:**
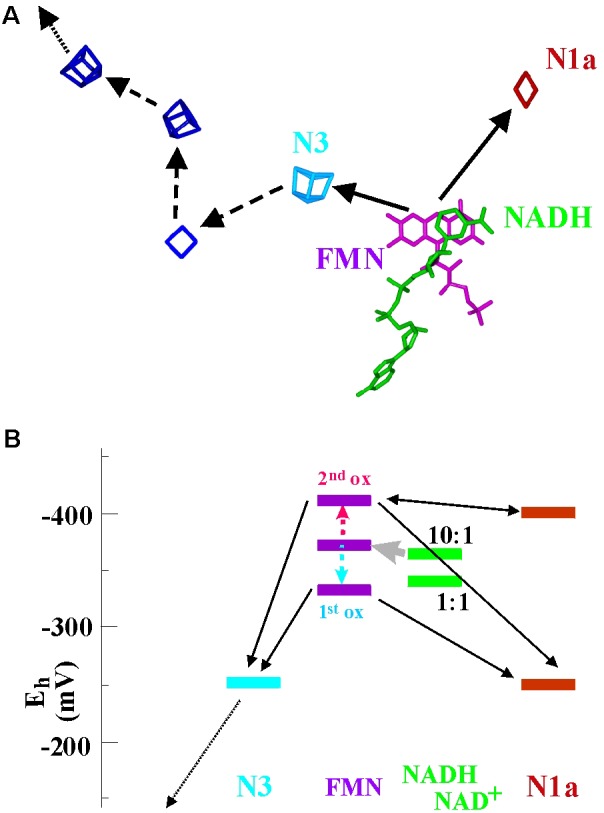
Spatial arrangement **(A)** and electrochemical landscape **(B)** of the NuoE/F-module in Complex I. The spatial bifurcation reaction is indicated in **(A)** by the two continuous arrows while the subsequent electron transfer events down the chain of iron–sulfur centers and toward the quinone (not shown) are marked by dashed and dotted arrows. In **(B)** two different redox potentials are depicted for cluster N1a, corresponding to the values reported for the mitochondrial and *Aquifex aeolicus* enzyme (–250 mV) and for the *Thermus thermophilus* enzyme (–400 mV).

NuoE and NuoF (also denoted Nqo1 and Nqo2) form a compact structural module (Sazanov, 2016) containing a flavin, a [4Fe-4S]- (“N3”) and a [2Fe-2S]-cluster (“N1a”). 3D structures with NAD in close proximity to the flavin have been obtained (**Figure 6A**) and the module is thought to negotiate between 2-electron oxidation of NADH and 1-electron reduction of the chain of iron–sulfur centers which leads toward the site of quinone reduction. The flavin in this case therefore acts as a gate between 2- and 1-electron redox compounds. Cluster N3 is the entry point to the electron wire to the quinone-binding site while cluster N1a appears as a dead end roughly opposite N3 with respect to the flavin moiety (**Figure 6A**) and has no obvious connection to other electron transfer chains. In Complex I from *Escherichia* (*E.*) *coli* and *Aquifex* (*A.*) *aeolicus*, cluster N1a features redox potentials of about -250 mV and is observed to go reduced upon addition of NADH to the enzyme. In the mitochondrial and the *Thermus thermophilus* enzymes it titrates close to -400 mV and does not seem to be reduced by NADH (Gnandt et al., 2016). Cluster N3 titrates in the vicinity of -250 mV in all systems (Gnandt et al., 2016). The flavosemiquinone was studied by EPR and a small but measurable logK_S_ of 1.34 was determined (Sled et al., 1994). This translates into crossed-over potentials with ΔE ∼-80 mV and individual potentials for the first and second oxidation reactions of the flavin at -336 and -414 mV, respectively. The electrochemical landscape resulting from these redox parameters is depicted in **Figure 6B**.

Obviously, this system lacks the endergonicity for the first electron leaving the flavin characteristic for canonical electron bifurcation, since it can flow downhill to cluster N3 (or also cluster N1a for the systems with E_m_ = -250 mV) with a drop in potential of almost 100 mV. The second electron can then exergonically reduce the remaining cluster or, for the case where E_m_ of N1a is around -400 mV, equilibrate between the flavosemiquinone and cluster N1a (**Figure 6B**). The two individual flavin oxidation events therefore aren’t coupled via the described escapement mechanism and no up-conversion of redox energy will take place. In agreement with these electrochemical properties, NADH-oxidation activity by quinone is only marginally affected when the redox potential of cluster N1a is mutationally shifted from -250 to -400 mV (Gnandt et al., 2016) and thus eliminated as an efficient electron acceptor. It therefore seems that the comparatively weak potential inversion of the flavin in the NuoE/F system allows for successive electron transfer down the wire through cluster N3 as well as for simultaneous reduction of the two iron–sulfur clusters N1a and N3. In the latter case the electrons bifurcate spatially but not thermodynamically. To put this in mechanistic terms, the gear-transmission-type coupling of the bifurcation reaction appears to have been replaced by a slip-clutch mechanism.

Intriguingly, the so-called “electron bifurcating hydrogenases” contain two subunits with substantial sequence similarity to NuoE and NuoF. As is in their name, these hydrogenases have been reported to bifurcate electrons derived from H_2_ oxidation toward reduction of NAD^+^ and ferredoxin. Enzymes of this type have been reported from several species, such as *Acetobacterium woodii* (Schuchmann and Müller, 2012), *Thermotoga maritima* (Schut and Adams, 2009), *Moorella thermoacetica* (Wang et al., 2013b), *Clostridium autoethanogenum* (Wang et al., 2013a), and *Ruminococcus albus* (Zheng et al., 2014). They all contain, in addition to the NuoE/F orthologs, an [Fe-Fe]-type hydrogenase subunit related to the monomeric [Fe-Fe]-hydrogenase of *Clostridium pasteurianum*. A subpopulation of these electron bifurcating hydrogenases furthermore contains a thioredoxin-like [Fe-S]-containing subunit which, however, does not seem essential for electron bifurcation (Poudel et al., 2016).

Like for many of the enzymes dealt with in this article, electron transfer direction is readily reversed in these hydrogenases and some of them thus also perform redox confurcation [e.g., the enzymes from *Moorella thermoacetica*, *Clostridium autoethanogenum*, and *Ruminococcus albus* (Wang et al., 2013a,b; Zheng et al., 2014)]. Unfortunately, no 3D structure has so far been solved for any of these enzymes. Based on the detection (Verhagen et al., 1999; Schut and Adams, 2009; Schuchmann and Müller, 2012; Wang et al., 2013b) of a single flavin-binding sequence motif (Hanukoglu and Gutfinger, 1989) in electron bifurcating hydrogenases, initially the presence of only one flavin molecule was proposed. The respective motif in the hydrogenases is at a sequence position equivalent to that of the motif in NuoE in our multiple sequence alignments. Putative ligands for two iron–sulfur centers corresponding to N1a and N3 in Complex I are also found in these alignments. More recently, Buckel and Thauer (2013) have postulated that “two FMN should be bound, one being required for NADH dehydrogenation and a second one for flavin-based electron bifurcation.” This reasoning makes perfect sense since two electrons need to be accumulated by a gating flavin to reduce NAD^+^ in a two-electron reaction, a task impossible to be achieved by the bifurcating flavin which sends its two electrons in opposite directions. The substantial sequence conservation between the two relevant subunits carrying the flavin and the N1a and N3 clusters and their homologs in the hydrogenases, together with the absence of any flagrant second flavin binding motif in both subunits, does not argue for the presence of a second flavin. This conundrum recently prompted the proposal that the bifurcating site in bifurcating hydrogenases may actually be at the active-site H-cluster rather than at the flavin (Peters et al., 2018).

Since in our view electron bifurcation arises from cooperative two-electron chemistry, the phenomenon likely isn’t restricted to redox-active organic molecules such as quinones and flavins but may be expected to also occur on transition metals or clusters thereof provided they feature three or more redox states and sufficiently strong positive redox cooperativity between the respective redox transitions. The case of molybdenum, for which all these properties have been observed empirically, will be discussed below. While the redox spacing between the two transitions (ferrous/ferric and ferric/ferryl) of mononuclear iron is generally found to be discouragingly wide (almost 1 V) and redox potential inversion hence unlikely, the electrochemical properties of the two-iron containing H-cluster may be more conducive to crossing of the individual potentials. However, if the 3D-structure of the [Fe-Fe]-hydrogenase from *Clostridium pasteurianum* (with almost 40% sequence similarity to the HydA subunit in the bifurcating enzyme from *Thermotoga maritima*) provides any guidance, we have difficulties seeing a potential second entry pathway (complementing the linear chain of [4Fe-4S]-clusters for electron confurcation) toward the H-cluster which is deeply buried within its parent protein subunit. Nevertheless, the proposed shift of the bifurcating site from the flavin to the H-cluster represents a novel approach to rationalize the enigma of bifurcating hydrogenases and calls for experimental scrutiny.

## Evolutionary Use of the Construction Kit

The observation that the overwhelming majority of the flavoenzymes involved in bioenergetic electron transfer seems to be put together from a very limited set of flavin-binding domains together with a host of other elementary, often redox-cofactor containing, building components also found in many other enzymes once more emphasizes the construction-kit principle employed by evolution in the generation of novel bioenergetic enzymes (Friedrich and Weiss, 1997; Baymann et al., 2003; Grein et al., 2013; Grimaldi et al., 2013; Magalon et al., 2016). Rather than evolving novel protein architectures, nature appears to mainly draw from a pre-existing reservoir of structural motifs. Interestingly, these motifs often, but not always, are correlated to specific functional features. For example, enzymes employing the so-called Molybdo/tungsto-*bis*PGD building component generally catalyze redox conversions of two-electron redox compounds present in the environment (Grimaldi et al., 2013), the membrane-integral diheme cytochromes interconnect lipo-soluble and water-soluble electron transfer chains (Schoepp-Cothenet et al., 2013) and tetracubane iron–sulfur proteins serve as electron wires (for further examples, see Baymann et al., 2003). This begs the question whether the differing structural motifs are correlated to the different positively or negatively cooperative two-electron transfer properties discussed above and indicated in **Figure 1**. In **Supplementary Table S1**, we have compiled electrochemical properties of flavin-cofactors in respective enzymes as reported in the literature.

The color-coding employed in **Figure 5** correlates the depicted structural modules to electron transfer function (that is, bifurcating and both-together in red/orange, gating in violet and one-by-one-type in blue). A more complete representation can be found in **Supplementary Figure S2** and **Supplementary Table S1**. A clear trend, albeit not a strict rule, emerges from this correlation which admittedly may be biased by limitations in the currently available set of 3D structures. So far, members of the NDH-2 family appear to predominantly perform either both-together or bifurcating electron transfer and accordingly feature strongly inverted potentials (in most cases evidenced by the absence of an observable flavo-semiquinone). The depicted cases structurally resembling FNR all mediate electron transfer between 2-electron and 1-electron redox centers. The group of flavodoxin folds encompasses flavoproteins featuring all types of electron transfer properties (both together, bifurcating, gating and single-electron).

The fact that some correlation between protein structure and electron transfer properties of the flavin cofactor can be observed is astonishing in the light of results obtained on FNR from the cyanobacterium *Anabaena*. Very subtle modifications of the hydrogen-bonding network involving the N_5_-nitrogen of the flavin moiety were found to induce drastic alterations in the relative redox midpoint potentials and to shift the individual potentials by about 100 mV further into the inverted region (Sánchez-Azqueta et al., 2014) which corresponds to a decrease of K_S_ by roughly two orders of magnitude. Given this subtle dependence of the strength of redox cooperativity on details of the hydrogen-bonding network surrounding the flavin, evolutionary changes likely would be able to easily redox-tune any of the discussed structural motifs to meet all kinds of electrochemical requirements. They indeed do so as for example in the enzyme dihydropyrimidine dehydrogenase (DPD) which features the canonical fold of the NDH-2 family but a mode of electron transfer falling into the realm of gating mechanisms. **Supplementary Figure S1** shows several further cases which feature similar folds but strongly dissimilar electron transfer properties. In many cases, however, evolution appears to prefer recruiting for a specific function the protein fold which already performed the respective task earlier. This observed tendency to use ready-made functional modules rather than *de novo* engineered protein units nicely illustrates François Jacob’s notion of evolution by tinkering (Jacob, 1977).

Two or three distinct flavin-containing folds can now be added to the construction kit for electron transfer enzymes, depending on whether we count the FNR fold as a family of its own right or as a complexified version of the structurally simpler flavodoxin motif. Conserved 3D structures are frequently interpreted to suggest common evolutionary origins and it was for this reason that we refer to groups of enzymes exhibiting related folds as “families” in this contribution. Consequently, the protein or gene sequences of the individual proteins in the families depicted in **Figure 3** may contain the imprints of their evolutionary history (Zuckerkandl and Pauling, 1965). Reconstruction of the phylogenetic tree of such a family may, under certain circumstances, allow the inference of evolutionary ancestry of the fold and consequently of its related function. This approach has been applied to Rieske/cyt*b* complexes which harbor the quinone-based Q_o_-site electron bifurcation reaction. Evolutionary roots reaching back to the last universal common ancestor (LUCA) of living beings were indeed derived from these analyses (Schütz et al., 2000; Lebrun et al., 2006; Ducluzeau et al., 2009; Ten Brink et al., 2013; Kao and Hunte, 2014). Several of the flavin enzyme families discussed in this article, such as ETF (Garcia Costas et al., 2017), Hdr or NDH-2 (Ojha et al., 2007; Marreiros et al., 2016), are widely distributed over both Bacteria and Archaea and their presence as early as in LUCA is therefore imaginable. A recently published comprehensive analysis of the phylogenetic distribution of ETF reconstructed an unrooted tree based on concatenated sequences (Garcia Costas et al., 2017). Although the lack of a reliable root precludes conclusions on pre- or post-LUCA origins of the observed clades of paralogs, the reported phylogeny does not exclude a pre-LUCA emergence of the entire enzyme superfamily. The Hdr enzyme from methanogens, for which a 3D structure has recently been obtained (Wagner et al., 2017), actually is part of a large superfamily with representatives in many organisms from both the archaeal and the bacterial domains (Grein et al., 2013). Hdr-related enzymes in the form of the Flx-HdrABC complex have notably been postulated to participate in sulfate-reducing bioenergetic pathways and to reduce ferredoxin and disulfides *via* electron bifurcation in *Desulfovibrio* species (Ramos et al., 2015). Hdr-related genes have furthermore been identified in several sulfur-oxidizing prokaryotes and a corresponding enzyme has been purified from the chemolithotrophic sulfur-oxidizer *Aquifex aeolicus* (Boughanemi et al., 2016). The phylogenetic relationships between all these Hdr-like entities remain so far only poorly understood. In general, phylogenetic approaches to the young field of flavin-based electron bifurcation are in their infancies and carry the potential for exciting future discoveries. In the absence of reliable top–down results, that is, conclusions derived from molecular phylogenies of enzymes involved in flavin-based electron bifurcation, we will in the following try to address the problem of the mechanism’s ancestry via plausibility arguments based on thermodynamics and palaeogeochemistry.

## On the Evolutionary Emergence of Electron Bifurcation

As argued already more than half a century ago (Schrödinger, 1944), the crucial property defining life is its ability to convert environmental disequilibria into intracellular disequilibria. This argument has since been elaborated to great detail (Cottrell, 1979; Branscomb and Russell, 2013, 2018; Branscomb et al., 2017). Frequently cited examples for extant biological processes performing this fundamental task comprise ATP synthases and the redox-driven ion pumps of bioenergetic systems. The general principle of action in these systems is that endergonic and exergonic partial reactions are strictly coupled so that the overall reaction, while being exergonic, is contingent upon the actual proceeding of the endergonic partial reaction (Branscomb et al., 2017), just as described above for electron bifurcation. Given the outstanding importance of electron bifurcation as an up-converter of redox disequilibria and therefore of a local increase in order, we consider that life could have taken advantage of this process from its very beginnings. Furthermore, since electron bifurcation relies on the redox properties of specific molecules rather than on the specific action of dedicated enzymes, the general mechanism may well have operated in nascent life prior to the emergence of catalysis based on large enzymatic machineries.

Free energy converting reactions performed by transition metals and clusters thereof have been discussed previously as fundamental processes in nascent life. Mineral-harbored metal clusters lend themselves as first catalysts since they are abundant in the geosphere and have versatile electron transfer and ligand binding properties (Nitschke et al., 2013). Electron bifurcation, however, has been discussed in this contribution as a mechanism based on organic molecules (quinones and flavins; however, see the final paragraph of the NuoEF-section) which may at first sight seem to exclude their contribution in nascent life.

We will in the following advocate that firstly, transition metals themselves can perform bifurcating electron transfer reactions just as quinones and flavins do and secondly, that flavins may have been among life’s earliest organic molecules. Tungsten and molybdenum are transition metals which readily perform two-electron transitions. Molybdenum is found in cofactors of various enzymes, notably in the form of molybdo/tungsto-pterins. Enzymes containing the Mo/W-pterin cofactor perform the redox conversion of a plethora of environmental substrates with two-electron chemistry (Grimaldi et al., 2013) and several of them likely are at least as old as the LUCA (Schoepp-Cothenet et al., 2012). The Mo-pterin cofactor in specific enzymes from the superfamily can adopt strongly positive cooperative redox behavior (Hoke et al., 2004; Duval et al., 2016), that is, feature substantially inverted potentials for its individual one-electron transitions (Duval et al., 2016). The Mo/W-pterin cofactor therefore possesses all the properties necessary for electron bifurcating reactions, although a Mo-pterin-based bifurcating enzyme has not been described so far.

These observations from extant biology lead us to propose a specific succession of events occurring at the putative habitats for life’s emergence, that is, submarine alkaline hydrothermal vents, and paving the way from minerals to the biological redox cofactor flavin. Intriguingly, occasional enrichments of molybdenum are found in the 3.8 billion years old Eoarchaean Banded Iron Formation (BIF) in Isua Greenland (Appel, 1980; Dymek and Klein, 1988). This is not so surprising given its solubility in reduced alkaline hydrothermal fluids, variously as [MoO_4_]^2-^, [MoS_4_]^2-^, [FeO(OH)MoS_4_]^3-^, and [(Fe_2_S_2_)(MoS_4_)_2_]^4-^ (Helz et al., 2013; Russell and Nitschke, 2017). These ions would precipitate at the vent on meeting the acidulous and reduced early ocean, as would the double layer oxyhydroxide green rust (GR, ∼[Fe^II^_6x_Fe^III^_6(1-x)_ O_12_H_2(7-3x)_]^2+^ [CO_3_.3.3H_2_O]^2-^) (Antony et al., 2008), the precursor to the iron oxide minerals presently constituting the BIF (Arrhenius, 2003; Russell et al., 2013; Tosca et al., 2016; Halevy et al., 2017; Russell, 2017). In these conditions the carbonates could have been replaced by the molybdate counter-ions proton-caged in the interlayer galleries where they may have enabled the various two-electron transfer processes now carried out by the molybdo-enzymes today (Itaya et al., 1987; Strajescu et al., 1997; Rives and Ulibarri, 1999; Evans and Slade, 2006; Wander et al., 2008; Duan et al., 2011; Duval et al., 2016).

**FIGURE 7:**
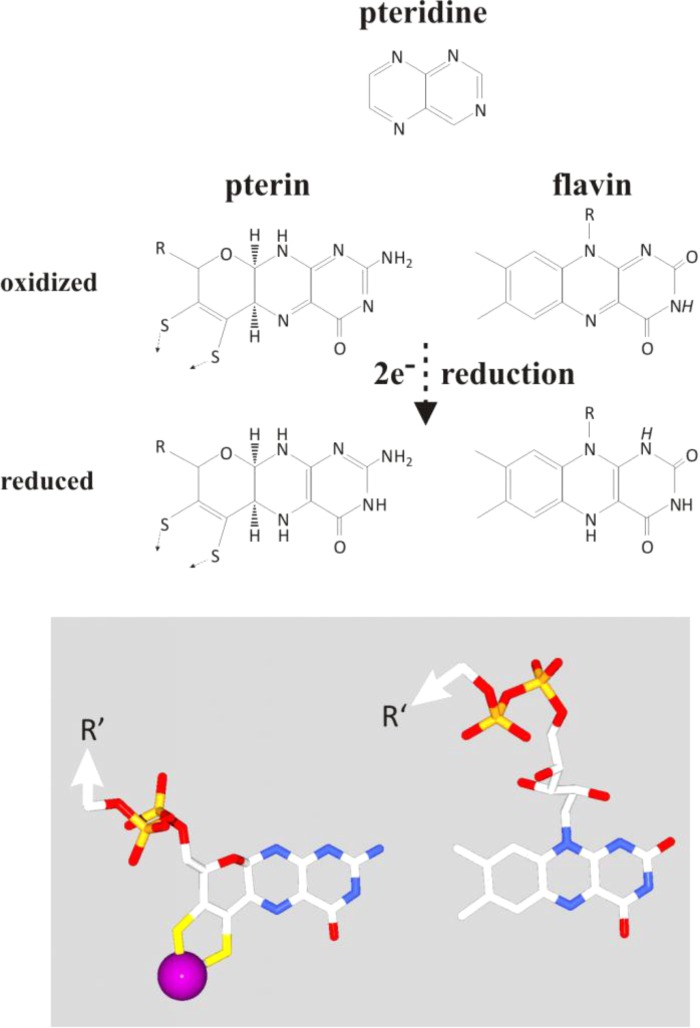
Comparison of the chemical layout (top part) and 3D-conformation (bottom part) of pterins and flavins together with a representation of their precursor-moiety, the pteridine (top). Protons indicated in italics on the flavin molecule feature pK-values in the physiological range of pH.

The early history of life was marked by the subjugation, sequestering and the partial dissolution of these mineral nanoengines. Upon the emergence of the first simple organic molecules, pteridines (synthesized today in Archaea and Bacteria from guanosine triphosphate, GTP) or their derivatives, the pterins, may have started to serve as ligands for molybdenum, thereby solubilizing the transition metals out of the green rust mineral and eventually yielding the molybdopterin cofactor of extant enzymes. When the first cells ventured, or were born by currents, beyond the alkaline waters into the wider Hadean or Archaean acidulous and reduced ocean, their survival would be challenged by the dearth of soluble molybdenum anions (Anbar, 2008; Boyd et al., 2011; Zhang et al., 2014). During this period of the organic takeover, pterins may have evolved into the chemically closely related flavins (Basu and Burgmayer, 2011) which mimic the electrochemical properties of the molybdopterins based only on the aromatic ring system. **Figure 7** illustrates the structural similarities and differences between pterins and flavins. The similarities mainly trace back to the pteridine moiety from which both pterins and flavins are derived while the third rings of the two systems differ in bond-saturation. This ring is fully conjugated and thus coplanar with the pteridine-part in flavins whereas its higher saturation due to the oxygen heteronucleus leads to substantial puckering highlighted by the 3D-representation at the bottom of the figure. Intriguingly, both pterins and flavins undergo two-electron oxidoreduction reactions (**Figure 7**). Noteworthily, even in extant molybdoenzymes, redox properties are strongly influenced by the H-bonding network surrounding the pterin moiety (Wu et al., 2015; Duval et al., 2016) just as is the case for isoalloxazine molecule in flavoenzymes. Thus, while molydopterin itself was retained for redox ranges inaccessible to the flavins, the flavoproteins themselves may have evolved to stand in for the absent molybdenum anion in many of its other former roles, especially perhaps in the redox bifurcation of electrons.

## Author Contributions

All authors listed have made a substantial, direct and intellectual contribution to the work, and approved it for publication.

## Conflict of Interest Statement

The authors declare that the research was conducted in the absence of any commercial or financial relationships that could be construed as a potential conflict of interest.

## Footnotes

1 According to the Nernst equation E_h_ = E_m_ + 2.3RT/nF^∗^log([ox]/[red] with E_h_, solution potential; E_m_, redox midpoint potential; n, number of transferred electrons; T, temperature in Kelvin; R, universal gas constant; F, Faraday constant; note that under specific conditions the more appropriate chemical activities “a” rather than concentrations “[c]” need to be considered).

2 K_S_ = [semi-reduced]^2^/[ox]x[red].

3 ^∗^: for a compilation of pertinent mathematical derivations and correlations as well as graphic representations thereof, see our dedicated website at http://bip.cnrs-mrs.fr/bip09/2electron.html.

## Supplementary Material

FIGURE S1 Structures of flavoenzymes containing at least one substrate molecule, substrate analog, or electron transfer partner of the flavin are superimposed on their flavin ring system (in gray in the figure). Substrates are found to be mainly located on the Re-side of the flavin (above the flavin in the figure) with their ring systems oriented parallel to the isoalloxazine moiety. Cofactors serving as electron transfer partners are mainly found on either side of the isoalloxazine ring and rarely on its Re- or Si-side (i.e., above or below the plane of the isoalloxazine moiety). These spatial preferences are independent of the electron transfer properties of the flavin as evidenced by the color-coding (purple for substrates and/or electron transfer partners of gating type enzymes and orange for both-together or bifurcating types). Pdb-entry codes for the structures used in this comparison: subunit F of Complex I (pdb: 3IAM, *Bos taurus*), dihydroorotate dehydrogenase B, FAD domain (pdb: 1EP3, *Lactococcus lactis*); dihydroorotate dehydrogenase B, FMN domain (pdb: 1EP3, *Lactococcus lactis*), sulfite-quinone reductase (pdb: 3HYW, *Aquifex aeolicus*), dihydropyrimidine dehydrogenase, FMN domain (pdb: 1GTE, *Sus scorfa*), dihydropyrimidine dehydrogenase, FAD domain (pdb: 1GTE, *Sus scorfa*), adenylsulfate reductase (pdb: 1JNR, *Archaeoglobus fulgidus*), NADH-dependent Ferredoxin:NADP oxidoreductase, small subunit (5JCA_S, *Thermotoga maritima*), heterodisulfide reductase (pdb: 5ODC, *Methanothermococcus*), NADH-dependent glutamate synthase alpha subunit, FMN domain (pdb: 2VDC, *Azospirillum brasilense*), NADH-dependent glutamate synthase alpha subunit, FAD domain (pdb: 2VDC), fumarate quinol oxidoreductase (1QLB, *Wolinella succinogenes*), NADH-dehydrogenase 2 (pdb 4NWZ, *Saccharomyces cerevisiae*), sarcosine oxidase (pdb: 1X31, *Corynebacterium* sp. U-96), Ferredoxin NADH oxidoreductase (pdb: 1QGA, *Pisum sativum*), Ferredoxin: ferredoxin NADH oxidoreductase complex (1EWY, *Anabaena* PCC7119), Na^+^-translocating NADH oxidoreductase (pdb: 4P6V, *Vibrio cholerae*).

FIGURE S2 Structures of diverse FAD- or FMN-containing enzymes. Only the flavin-binding domains are depicted. The flavin is in red. Shades of orange were used for enzymes that perform both-together electron transfer or electron bifurcation, shades of violet for enzymes that perform the gating-type electron transfer and blue for enzymes that transfer only a single electron. The enzymes are classified according to their fold. See text for a more detailed description. NDH-2, NADH-dehydrogenase 2 (pdb 4NWZ); SQR, sulfite-quinone reductase (pdb: 3HYW); Nfn-1 (L), NADH-dependent Ferredoxin:NADP oxidoreductase, large subunit (5JCA,L); Hdr, heterodisulfide reductase (pdb: 5ODC); TrxR, thioredoxin reductase (pdb: 1f6m); DPD, dihydropyrimidine dehydrogenase (pdb: 1GTE); GR-FNR, glutathione reductase-type NADH-ferredoxin oxidoreductase (pdb: 2LZX); GRT, glutathione reductase (pdb: 2GRT); NROR, NADH rubredoxin reductase (pdb: 3KLJ); AprA, adenylsulfate reductase (pdb: 1JNR); Etf:Q, electron-transfer flavoprotein-ubiquinone oxidoreductase (pdb: 2GMJ); CII (Complex II), succinate quinone oxidoreductase (1QLB); SO, sarcosine oxidase (pdb: 1X31); FNR, ferredoxin NADH oxidoreductase (pdb: 1QUE); Nqr, Na^+^-translocating NADH oxidoreductase (pdb: 4P6V); sMMO, soluble methane monooxygenase (pdb: 1TVC); Nfn-1 (S), NADH-dependent Ferredoxin:NADP oxidoreductase, small subunit (5JCA,S); b5r, cytochrome b5 reductase (pdb: 1IBO); PDR, phthalate dioxygenase reductase (pdb: 2PIA); Fld, flavodoxin (pdb: 2WC1); FIRd, flavo-diiron nitric oxide reductase (pdb: 5LMC); ETF-α, electron-transfer-flavoprotein, alpha subunit (pdb: 2L2I); ETF-β, Electron Transfer Flavoprotein, beta subunit (pdb: 2L2I); DHOdhA_FMNa, dihydroorotate dehydrogenase A, N-ter FMN domain (pdb: 1DOR); DHOdhA_FMNb, dihydroorotate dehydrogenase A, C-ter FMN domain (pdb: 1DOR); DHOdhB_FAD, dihydroorotate dehydrogenase B, FAD domain (pdb: 1EP3); DHOdhB_FMN, dihydroorotate dehydrogenase B, FMN domain (pdb: 1EP3); Fd-Glts, glutamate synthase (pdb: 1OFE); Glts α NADH-dependent glutamate synthase alpha subunit (pdb: 2VDC); Flcytb2, flavocytochrome b2 (pdb: 1FCB); IYD, iodo-tyrosine deiodinase (pdb: 4TTC); Nfr, nitro-flavin-reductase (pdb: 3N2S); Bcd, butyryl CoA dehydrogenase (pdb: 1BUC); SCAD, short chain acyl-CoA dehydrogenase (pdb: SCAD); PyrOx, pyruvate oxidase (pdb: 4FEG); Q-PyrOx, quinone pyruvate oxidase (pdb: 3EY9); NuoF, subunit F of Complex I (pdb: 3IAM).

TABLE S1 The table represents a compilation of flavoproteins for which electrochemical properties are available in the literature and a comparison to the relevant redox potentials of free FAD and FMN as well as of the soluble 2-electron redox substrate NAD(P). The color-coding in the column labeled “reaction” is detailed at the bottom of the table and corresponds to the nomenclature for differing types of 2-electron redox reactions as used throughout the main text of this article.
