# Supplementary figures and images for: On the Natural History of Flavin-Based Electron Bifurcation
Source: Front Microbiol. 2018 Jul 3;9:1357. doi: 10.3389/fmicb.2018.01357 (PMC6037941; doi:10.3389/fmicb.2018.01357)

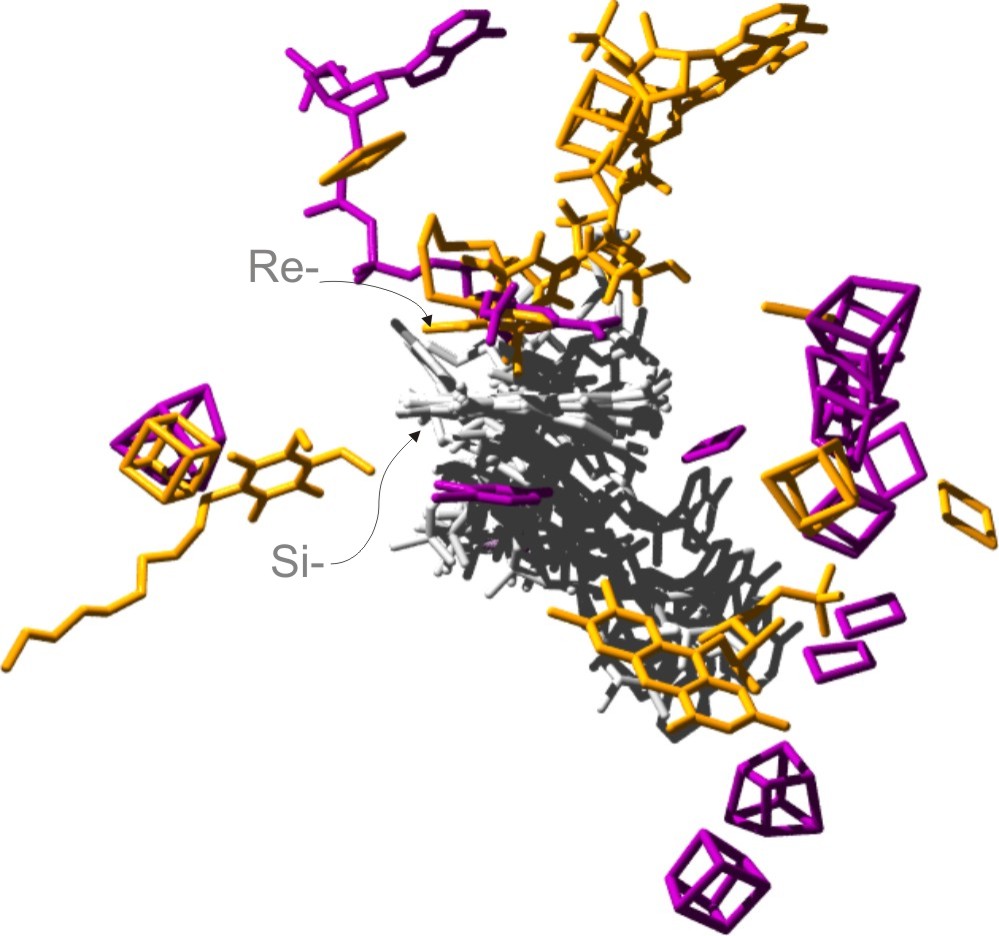

Supplement: FIGURE S1 — Structures of flavoenzymes containing at least one substrate molecule, substrate analog, or electron transfer partner of the flavin are superimposed on their flavin ring system (in gray in the figure). Substrates are found to be mainly located on the Re-side of the flavin (above the flavin in the figure) with their ring systems oriented parallel to the isoalloxazine moiety. Cofactors serving as electron transfer partners are mainly found on either side of the isoalloxazine ring and rarely on its Re- or Si-side (i.e., above or below the plane of the isoalloxazine moiety). These spatial preferences are independent of the electron transfer properties of the flavin as evidenced by the color-coding (purple for substrates and/or electron transfer partners of gating type enzymes and orange for both-together or bifurcating types). Pdb-entry codes for the structures used in this comparison: subunit F of Complex I (pdb: 3IAM, Bos taurus), dihydroorotate dehydrogenase B, FAD domain (pdb: 1EP3, Lactococcus lactis); dihydroorotate dehydrogenase B, FMN domain (pdb: 1EP3, Lactococcus lactis), sulfite-quinone reductase (pdb: 3HYW, Aquifex aeolicus), dihydropyrimidine dehydrogenase, FMN domain (pdb: 1GTE, Sus scorfa), dihydropyrimidine dehydrogenase, FAD domain (pdb: 1GTE, Sus scorfa), adenylsulfate reductase (pdb: 1JNR, Archaeoglobus fulgidus), NADH-dependent Ferredoxin:NADP oxidoreductase, small subunit (5JCA_S, Thermotoga maritima), heterodisulfide reductase (pdb: 5ODC, Methanothermococcus), NADH-dependent glutamate synthase alpha subunit, FMN domain (pdb: 2VDC, Azospirillum brasilense), NADH-dependent glutamate synthase alpha subunit, FAD domain (pdb: 2VDC), fumarate quinol oxidoreductase (1QLB, Wolinella succinogenes), NADH-dehydrogenase 2 (pdb 4NWZ, Saccharomyces cerevisiae), sarcosine oxidase (pdb: 1X31, Corynebacterium sp. U-96), Ferredoxin NADH oxidoreductase (pdb: 1QGA, Pisum sativum), Ferredoxin: ferredoxin NADH oxidoreductase complex (1EWY, Anabaena PCC7119), Na+-translo [file Image_1.JPEG]

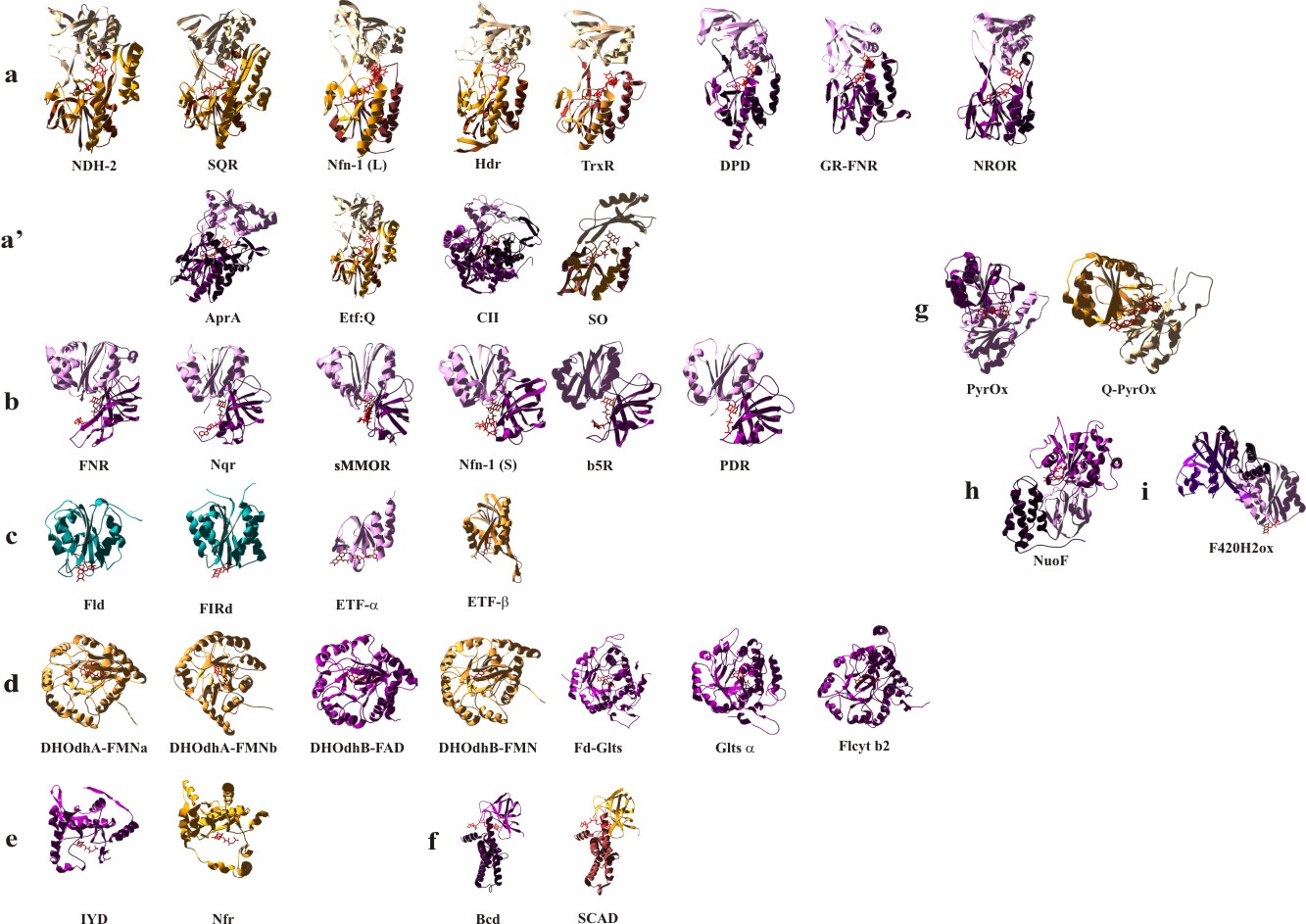

Supplement: FIGURE S2 — Structures of diverse FAD- or FMN-containing enzymes. Only the flavin-binding domains are depicted. The flavin is in red. Shades of orange were used for enzymes that perform both-together electron transfer or electron bifurcation, shades of violet for enzymes that perform the gating-type electron transfer and blue for enzymes that transfer only a single electron. The enzymes are classified according to their fold. See text for a more detailed description. NDH-2, NADH-dehydrogenase 2 (pdb 4NWZ); SQR, sulfite-quinone reductase (pdb: 3HYW); Nfn-1 (L), NADH-dependent Ferredoxin:NADP oxidoreductase, large subunit (5JCA,L); Hdr, heterodisulfide reductase (pdb: 5ODC); TrxR, thioredoxin reductase (pdb: 1f6m); DPD, dihydropyrimidine dehydrogenase (pdb: 1GTE); GR-FNR, glutathione reductase-type NADH-ferredoxin oxidoreductase (pdb: 2LZX); GRT, glutathione reductase (pdb: 2GRT); NROR, NADH rubredoxin reductase (pdb: 3KLJ); AprA, adenylsulfate reductase (pdb: 1JNR); Etf:Q, electron-transfer flavoprotein-ubiquinone oxidoreductase (pdb: 2GMJ); CII (Complex II), succinate quinone oxidoreductase (1QLB); SO, sarcosine oxidase (pdb: 1X31); FNR, ferredoxin NADH oxidoreductase (pdb: 1QUE); Nqr, Na+-translocating NADH oxidoreductase (pdb: 4P6V); sMMO, soluble methane monooxygenase (pdb: 1TVC); Nfn-1 (S), NADH-dependent Ferredoxin:NADP oxidoreductase, small subunit (5JCA,S); b5r, cytochrome b5 reductase (pdb: 1IBO); PDR, phthalate dioxygenase reductase (pdb: 2PIA); Fld, flavodoxin (pdb: 2WC1); FIRd, flavo-diiron nitric oxide reductase (pdb: 5LMC); ETF-α, electron-transfer-flavoprotein, alpha subunit (pdb: 2L2I); ETF-β, Electron Transfer Flavoprotein, beta subunit (pdb: 2L2I); DHOdhA_FMNa, dihydroorotate dehydrogenase A, N-ter FMN domain (pdb: 1DOR); DHOdhA_FMNb, dihydroorotate dehydrogenase A, C-ter FMN domain (pdb: 1DOR); DHOdhB_FAD, dihydroorotate dehydrogenase B, FAD domain (pdb: 1EP3); DHOdhB_FMN, dihydroorotate dehydrogenase B, FMN domain (pdb: 1EP3); Fd-Glts, glutamate synthase (pdb [file Image_2.JPEG]
